# Key traveller groups of relevance to spatial malaria transmission: a survey of movement patterns in four sub-Saharan African countries

**DOI:** 10.1186/s12936-016-1252-3

**Published:** 2016-04-12

**Authors:** John M. Marshall, Mahamoudou Touré, André Lin Ouédraogo, Micky Ndhlovu, Samson S. Kiware, Ashley Rezai, Emmy Nkhama, Jamie T. Griffin, T. Deirdre Hollingsworth, Seydou Doumbia, Nicodem J. Govella, Neil M. Ferguson, Azra C. Ghani

**Affiliations:** Department of Infectious Disease Epidemiology, MRC Center for Outbreak Analysis and Modelling, Imperial College London, London, UK; Divisions of Biostatistics and Epidemiology, School of Public Health, University of California, Berkeley, CA USA; Malaria Research and Training Center, University of Bamako, Bamako, Mali; Centre National de Recherche et de Formation sur le Paludisme, Ouagadougou, Burkina Faso; Institute for Disease Modeling, Bellevue, WA USA; Chainama College of Health Sciences, Lusaka, Zambia; Environmental Health and Ecological Sciences Thematic Group, Ifakara Health Institute, Dar es Salaam, Tanzania; School of Life Sciences, University of Warwick, Warwick, Coventry, UK

**Keywords:** *Plasmodium falciparum*, Spatial transmission, Cluster analysis, Women with children, Youth workers, Mobile phones, Mali, Burkina Faso, Zambia, Tanzania

## Abstract

**Background:**

As malaria prevalence declines in many parts of the world due to widescale control efforts and as drug-resistant parasites begin to emerge, a quantitative understanding of human movement is becoming increasingly relevant to malaria control. However, despite its importance, significant knowledge gaps remain regarding human movement, particularly in sub-Saharan Africa.

**Methods:**

A quantitative survey of human movement patterns was conducted in four countries in sub-Saharan Africa: Mali, Burkina Faso, Zambia, and Tanzania, with three to five survey locations chosen in each country. Questions were included on demographic and trip details, malaria risk behaviour, children accompanying travellers, and mobile phone usage to enable phone signal data to be better correlated with movement. A total of 4352 individuals were interviewed and 6411 trips recorded.

**Results:**

A cluster analysis of trips highlighted two distinct traveller groups of relevance to malaria transmission: women travelling with children (in all four countries) and youth workers (in Mali). Women travelling with children were more likely to travel to areas of relatively high malaria prevalence in Mali (OR = 4.46, 95 % CI = 3.42–5.83), Burkina Faso (OR = 1.58, 95 % CI = 1.23–1.58), Zambia (OR = 1.50, 95 % CI = 1.20–1.89), and Tanzania (OR = 2.28, 95 % CI = 1.71–3.05) compared to other travellers. They were also more likely to own bed nets in Burkina Faso (OR = 1.77, 95 % CI = 1.25–2.53) and Zambia (OR = 1.74, 95 % CI = 1.34 2.27), and less likely to own a mobile phone in Mali (OR = 0.50, 95 % CI = 0.39–0.65), Burkina Faso (OR = 0.39, 95 % CI = 0.30–0.52), and Zambia (OR = 0.60, 95 % CI = 0.47–0.76). Malian youth workers were more likely to travel to areas of relatively high malaria prevalence (OR = 23, 95 % CI = 17–31) and for longer durations (mean of 70 days *cf* 21 days, p < 0.001) compared to other travellers.

**Conclusions:**

Women travelling with children were a remarkably consistent traveller group across all four countries surveyed. They are expected to contribute greatly towards spatial malaria transmission because the children they travel with tend to have high parasite prevalence. Youth workers were a significant traveller group in Mali and are expected to contribute greatly to spatial malaria transmission because their movements correlate with seasonal rains and hence peak mosquito densities. Interventions aimed at interrupting spatial transmission of parasites should consider these traveller groups.

**Electronic supplementary material:**

The online version of this article (doi:10.1186/s12936-016-1252-3) contains supplementary material, which is available to authorized users.

## Background

As a vector-borne disease, malaria is spread alternately between its human and mosquito hosts. Humans travel much larger distances than mosquitoes and consequently human movement plays a dominant role in parasite dispersal [1, 2]. Significant funding is currently being invested in global malaria control [3] and as transmission declines [3, 4], a quantitative understanding of human movement is important to determine how best to target interventions [2]. Quantifying the dispersal of malaria parasites is particularly relevant once overall transmission has declined because, in combination with knowledge of environmental heterogeneity, it allows sources and sinks of transmission to be identified. Control programmes can then be designed that target the ‘hot spots’ and ‘hot pops’ of transmission [5–7]. Human movement is also of relevance to the spread of drug-resistant malaria parasites, which have recently emerged in Southeast Asia [8].

Despite the importance of human movement data in planning disease control, significant data gaps remain [9], particularly in sub-Saharan Africa, where over 90 % of malaria-related deaths occur [3]. One new data source that is increasingly being used as a proxy for human movement is anonymous mobile phone signal data [2, 10, 11]. This is a powerful and comprehensive data source that can conceivably be obtained at large scale. However, one drawback is that it may give a biased estimate of movement patterns in many African countries where affluent men are more likely to be mobile phone owners [12], phone sharing is common among rural women, and many individuals use multiple SIM cards due to non-overlapping provider coverage [13]. An additional limitation is that young children rarely have mobile phones, but are the demographic among which malaria is most prevalent. Data on child movement are also often lacking in household surveys [14], where the number of trips away in the last year is measured without information on children accompanying family members.

Substantial qualitative work has been conducted describing the types of human movements in Africa, many of which are relevant to malaria transmission. For example, in the Sahel, a semi-arid region beneath the Sahara Desert including parts of Mali and Burkina Faso, seasonal rains are correlated with both agricultural labour movements and malaria transmission due to an abundance of vector-breeding sites [5, 15]. Rural youths tend to leave their villages after a season’s harvest to look for casual work in nearby cities, stay away for several months and then return to help with farm work in the next agricultural season [16–18]. There is also a strong ethnic dimension to movements—for instance, the Songhai in Mali are known as good traders, setting up shops in cities throughout the country and migrating seasonally to sell goods [19]. In East and Southern Africa, there is a strong culture of sending young children to boarding school [20], which is a source of youth circulation. Across the continent, migration to urban areas is becoming increasingly attractive for all ethnic groups as a source of employment, education and permanent settlement [21, 22], which itself leads to increased short-term visits from other family members [23].

**Fig. 1:**
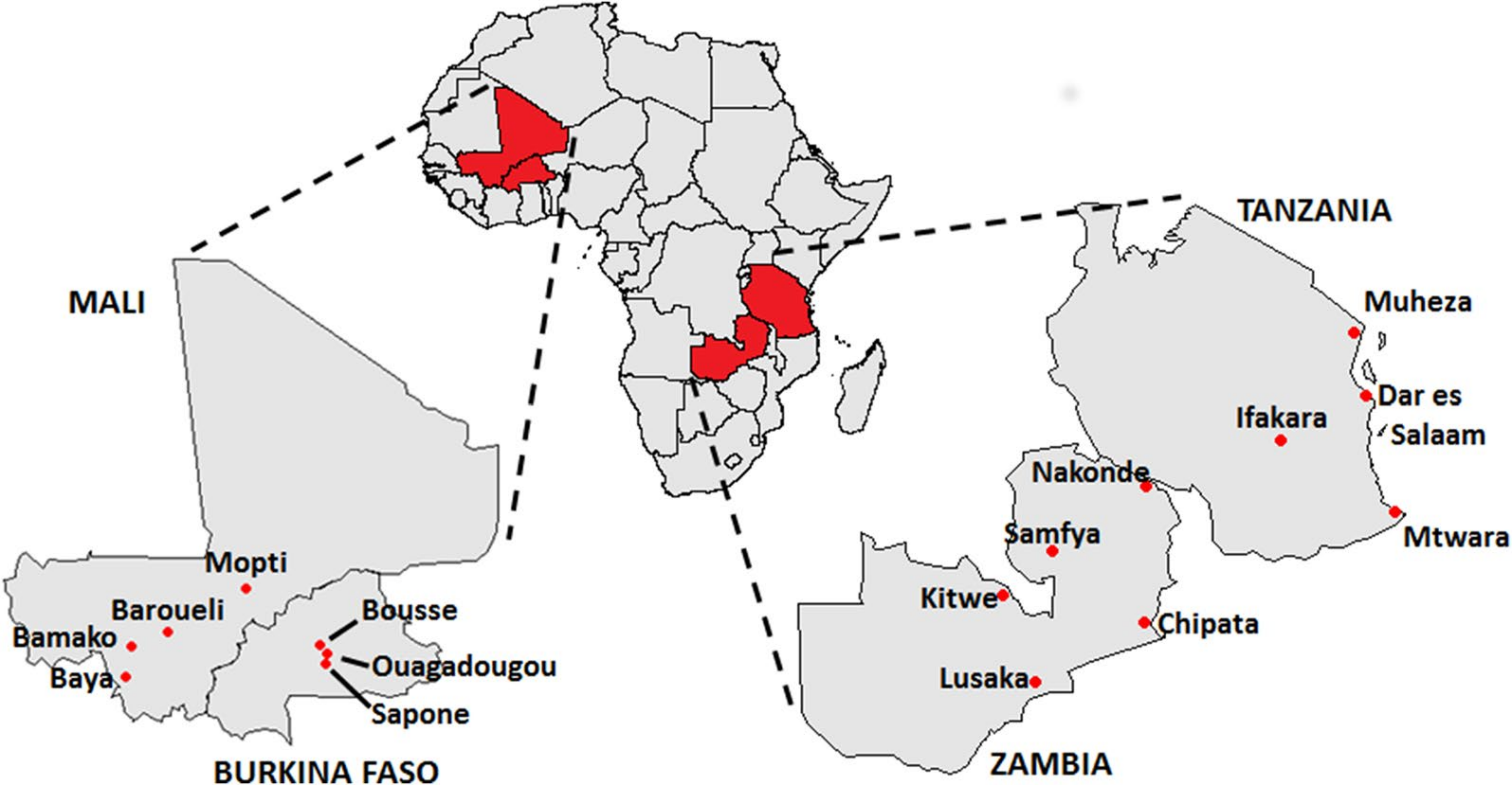
Survey locations in Mali, Burkina Faso, Zambia and Tanzania

To gain a better quantitative understanding of these patterns, a human movement survey was conducted in four countries with ongoing malaria transmission: two in West Africa (Mali and Burkina Faso), one in East Africa (Tanzania) and one in Southern Africa (Zambia) (Fig. 1). In consultation with local researchers, three to five survey locations were chosen in each country that were expected to capture a wide range of traveller groups. Survey respondents were asked about trips for which they had spent at least one night away from home, since the main African malaria vectors, *Anopheles gambiae* and *Anopheles funestus*, bite at night. Questions were also asked about demography and trip details to gain a better descriptive understanding of movement, about mobile phone usage to enable phone signal data to be better correlated with self-reported movement patterns, and about malaria risk behaviour and children accompanying travellers, since children are the most common parasite carriers.

## Methods

### Study sites

The survey was undertaken in four sub-Saharan African countries with ongoing malaria transmission—Mali, Burkina Faso, Zambia, and Tanzania (Fig. 1). In Mali and Burkina Faso, seasonal agricultural labour movements are common [15]. These countries are representative of a number of Sahelian countries in West Africa, including Senegal, Niger and Benin. These populations are highly dependent on arable land, with 80 % of the Malian labour force involved in fishing or agricultural activities [24] and 90 % of Burkinabe labour force involved in agriculture [24]. To explore the impact of survey timing on results, the Mali survey was conducted during consecutive rainy and dry seasons. Budgetary constraints did not permit this for other countries. Zambia and Tanzania both have a comprehensive national transport network, allowing their populations to be mobile on a large scale [24]. The population of both countries is also highly dependent on arable land; however, here agriculture is more stable year-round, leading to less seasonal movements [24].

The survey sites were chosen in collaboration with local malaria researchers and medical anthropologists. These were sites that: (a) were judged to capture a wide range of traveller groups present in the country; and, (b) had an existing relationship with the local researchers. Sampling of households within each study site was randomized, however, the study sites themselves were a judgement/convenience sample. Hence, inferences based on these results reflect the collection of study sites rather than the countries at large.

The Mali survey was conducted in Bamako, the capital city and largest urban centre, two fishing villages in Baya, two farming villages in Baroueli, and in Mopti and Fatoma, a commercial centre and village, respectively, approximately 460 km northeast of Bamako. The Burkina Faso survey was conducted in Ouagadougou, the capital and largest city, and two nearby locations—Sapone, an agricultural village approximately 50 km south of Ouagadougou, and Boussé, a major centre of agriculture and trade approximately 50 km north of Ouagadougou. The Zambia survey was conducted in five locations—Lusaka, the capital and largest city; Samfya, a central fishing town; Kitwe, an urban trading town in the Copperbelt; Nakonde, a town in the northeast bordering Tanzania; and, Chipata, a rural town in the east bordering Malawi. The Tanzania survey was conducted in four locations—Dar es Salaam, the capital and largest city; Ifakara, a small rural town on the edge of the Kilombero valley; Muheza, a small rural town near the Indian Ocean and the border with Kenya; and, Mtwara, an agricultural town with a growing mining industry near the Indian Ocean and Mozambique.

### Survey questions

Participants were recruited within each location using random sampling techniques (Additional file 1) and EpiCollect 2.0 as a data collection interface [25]. Participants were eligible for inclusion if they were 16 years of age or older and reported making at least one overnight trip in the last year. This parallels a question asked in the nationally representative Demographic and Health surveys (DHS) [14]. This allowed the survey to include a higher overall number of respondents providing information on travel patterns. The DHS survey data may be used to obtain an overall estimate of the frequency of travel and the characteristics of non-travellers.

Participants were asked a series of questions about short- and long-term circular movements over the last year (purpose, duration, month of departure, number of accompanying children on each trip), basic demographic information (age, gender, number of children under the age of five), mobile phone usage (ownership, reception, frequency of carriage, usage of a mobile phone), and malaria (perceived risk of malaria, bed net ownership, usage). Questions were also asked about migratory patterns. Details are provided in Additional file 1. The questionnaire was prepared in English, but administered in the languages of the local population.

A trip was considered short-term if its duration was 2 weeks or less, and long-term if its duration was longer than 2 weeks but less than a year. Details were recorded for the three most recent short- and long-term trips. Travel within the ward, commune or city of origin was not included. Destinations were recorded at the level of commune or ward via dropdown lists in Epicollect. Dropdown lists were populated using shape files for each of the study countries, allowing locations to be geocoded. Study participants were interviewed in Mali during the rainy season of September/October 2010 and the dry season of March 2011, in Burkina Faso during the rainy season of July 2011, in Zambia during the cool dry season of July/August 2012, and in Tanzania during the long rainy season of March 2013.

### Ethics

Ethical approval for the study was granted by the Imperial College Research Ethics Committee, UK and the Institutional Review Boards of the Malaria Research and Training Center in Mali, the *Centre National de Recherche et de Formation sur le Paludisme* in Burkina Faso, ERES Converge in Zambia and the Ifakara Health Institute Research Ethics Board in Tanzania. The survey was anonymous and informed consent was obtained from all participants.

### Statistical analysis

A descriptive analysis of the demographic data, trip properties, mobile phone usage, behaviour, and malaria-related variables was undertaken using individual trips as the unit of analysis. Hierarchical cluster analysis was performed using trip properties (logarithm of distance travelled, trip duration, season of departure, purpose, whether children accompanied, whether the origin and destination were rural or urban) and the demographics of the travellers (age, gender, number of children under 5 years, number of trips in the last year) as input variables in the FactoMineR package in R [26]. All recorded trips were included in the analysis. On average, 1.47 trips were analysed per person; however within-person correlation was not accounted for. As both continuous and categorical variables were analysed, the continuous variables were scaled to unit variance and the categorical variables were transformed into a disjunctive data table and scaled using multiple correspondence analysis. Missing values were replaced by the mean of the respective variable. The cluster analysis was performed both on the overall dataset and separately by country. Clusters were retained if they explained a significant amount of the variance in the combined analysis, determined if an additional cluster was associated with an inertia gain of *Q* > 0.5 [26].

Multivariable logistic regression was used to identify differences in mobile phone usage behaviour and malaria-related variables between the identified trip clusters. Malaria-related variables included bed net ownership, bed net usage at the origin and destination of travel, perceived malaria risk, and estimated malaria prevalence at the origin and destination of travel. Perceived malaria risk was derived from the following survey questions—“Do you think you are at risk of getting malaria where you live?” and “Do you perceive there to be a risk of malaria where you travelled to?” with high, medium and low response options. Estimated malaria prevalence was based on spatially stratified 2010 estimates of parasite prevalence in 2–10 years old [27] aggregated to the ward/commune level or to the next highest administrative level at which the origin and destination were resolved. Mobile phone-related variables included phone ownership, which was included in the full logistic regression model, and the frequency of carrying and using a phone, travelling with the phone, reception issues and phone sharing behaviour, which were analysed as part of a secondary logistic regression model conditional upon phone ownership.

## Results

### Survey results

In total, 4352 individuals were interviewed—1588 from Mali, 721 from Burkina Faso, 1093 from Zambia and 950 from Tanzania (Additional file 2). Table 1 summarizes the demographics of those surveyed. These reflect the random sample of people who made at least one overnight trip in the last year from the judgement/convenience sample of survey sites in each country. The Mali sample had significantly more male interviewees (854 males *cf* 730 females, p = 0.002), while the Zambia sample had significantly more female interviewees (671 females *cf* 413 males, p < 0.001). Interviewees in the Tanzanian sample reported more children under the age of five residing in the same residence than in the other countries (mean of 1.56 children *cf* 0.85 for other countries, p < 0.001) and interviewees in the Zambia sample reported more children under the age of five than in the Mali and Burkina Faso samples (mean of 1.00 children *cf* 0.79 for Mali/Burkina Faso, p < 0.001). Interviewees in the Burkina Faso and Zambia samples reported significantly more trips than interviewees in the Mali and Tanzania samples (mean of 2.75 trips *cf* 1.50 trips, p < 0.001), while DHS surveys reported a higher proportion of the populations of Zambia and Tanzania as having travelled in the last year compared to the populations of Mali and Burkina Faso [14, 28–30]. Bed net ownership was significantly lower among interviewees in the Zambian sample compared to the Tanzanian and Burkinabe sample (71 % ownership *cf* 86 % for Tanzania/Burkina Faso, p < 0.001). Mobile phone ownership was significantly lower among interviewees in the Malian sample compared to the Burkinabe and Tanzanian sample (54 % ownership *cf* 75 % for Tanzaznia/Burkina Faso, p < 0.001). Differences between the rainy and dry season samples in Mali are described in Section 2.1 of Additional file 1 and in Additional file 3: Table S1.

**Table 1:** Descriptive statistics of interviewees in each national sample

	Mali		Burkina Faso		Zambia		Tanzania	
	*N*	% (CI)	*N*	% (CI)	*N*	% (CI)	*N*	% (CI)
Total	1588		721		1093		950	
Gender								
Female	730	46 (43–48)	349	48 (45–52)	671	61 (58–64)	473	50 (47–53)
Male	854	54 (51–56)	365	51 (47–54)	413	38 (35–41)	470	49 (46–53)
Age								
16–29	760	48 (45–50)	316	44 (40–48)	482	44 (41–47)	416	44 (41–47)
30–45	511	32 (30–35)	284	39 (36–43)	395	36 (33–39)	381	40 (37–43)
>45	317	20 (18–22)	118	16 (14–19)	206	19 (17–21)	147	16 (13–18)
Mean number children <5 years (range)		0.81 (0–9)		0.77 (0–5)		1.01 (0–7)		1.56 (0–5)
Mean number trips (range)		1.56 (1–51)		3.42 (1–100)		2.30 (1–100)		1.40 (1–20)
Proportion who travelled (DHS)		0.313		0.291		0.431		0.427
Own bed net								
Yes	N/A	N/A	581	81 (78–83)	778	71 (68–74)	853	90 (88–92)
No	N/A	N/A	136	19 (16–22)	314	29 (26–32)	91	10 (8–12)
Own mobile phone								
Yes	859	54 (52–57)	527	73 (70–76)	715	65 (63–68)	726	76 (74–79)
No	727	46 (43–48)	191	26 (23–30)	330	30 (27–33)	217	23 (20–26)

The number of individuals (*N*) and percentage of the sample (exact binomial 95 % confidence interval), or mean (range) are shown. All interviewees did not answer all questions, hence there are some missing values

### Trip clusters

**Fig. 2:**
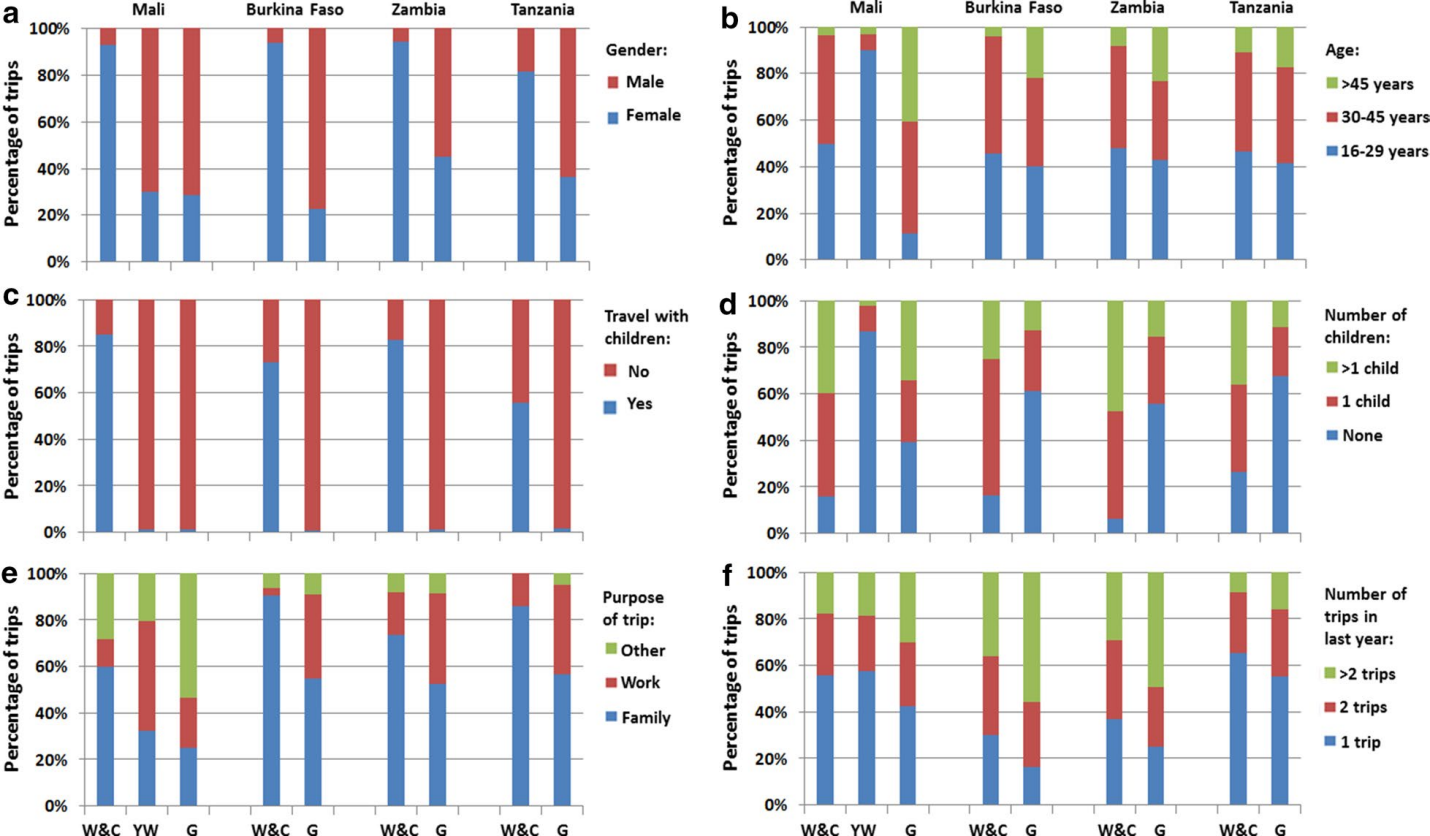
Demographic and trip characteristics of trip clusters. *Bar plot* representing percentages of demographic and trip variables by country and trip cluster (*W&C* women travelling with children, *YW* youth workers, *G* general cluster). **a** Gender; **b** age group; **c** children accompanying traveller or not; **d** number of children the traveller has age 5 years or under; **e** purpose of the trip; **f** Number of trips in last year

A cluster analysis identified three clusters of trips in the Mali sample and two in the Burkina Faso, Zambia and Tanzania samples based on the demographic and trip variables described in Methods. In all settings, the cluster that captured the greatest amount of variance in the data consisted predominantly of trips involving women between the ages of 16 and 45 years who travelled with children, usually for family-related reasons. This trip cluster is denoted as ‘women with children’ (Fig. 2) or ‘women travelling with children’ since the cluster is dominated by trips involving women travelling with children, although the clustering algorithm also included a smaller number of trips made by other groups. In the Mali, Burkina Faso and Zambia samples, 93–94 % of the trips in this cluster were made by women, 85, 73 and 83 % of whom were travelling with children, respectively. In the Tanzania sample, the cluster was less well defined, with 82 % of the trips being made by women, 56 % of whom travelled with children. In all four country samples, <1 % of the trips in the remaining clusters involved travel with children. The ‘women with children’ trip cluster also emerged in a combined, four-country cluster analysis and captured the greatest amount of variance in the data in this analysis.

**Fig. 3:**
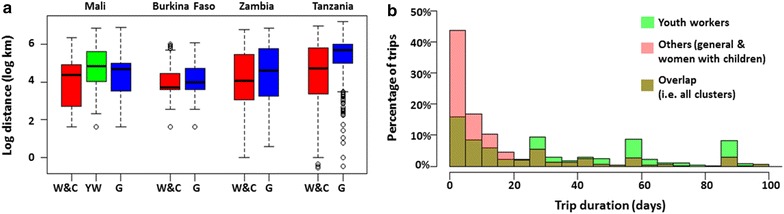
Distance and duration distributions of trip clusters. **a** *Box plot* of log trip distances for the different trip clusters (*W&C* women travelling with children, *YW* youth workers, *G* general cluster) by country. Median lines represent the 50th percentile and box edges represent the 25th and 75th percentile of log trip distance. *Circles* represent outliers, and the *lines* outside the *boxes* represent the range of log trip distance excluding outliers. **b** Histogram of trip durations for youth workers and others (general and women travelling with children) for Mali

Trips in the ‘women with children’ cluster tended to involve shorter distances than other clusters (mean of 136 km *cf* 191 km for other clusters, p < 0.001). For example, in the Mali sample, the mean trip distance for trips in the ‘women with children’ cluster was 120 km, compared to 157 km for those in other clusters, and in the Zambia sample, the mean trip distance was 161 km for the ‘women with children’ cluster, compared to 203 km for other trip clusters (Fig. 3a). Travellers in the ‘women with children’ cluster also tended to make fewer trips than those in other clusters (mean of 2.2 trips *cf* 3.3 trips for other clusters, p < 0.001). For example, in the Burkina Faso sample, travellers in the ‘women with children’ cluster made a mean of 2.5 trips in the last year, compared to travellers in other clusters who made a mean of 5.7 trips (Fig. 2f).

In the Mali sample, the cluster of trips that captured the second highest amount of variance in the data consisted predominantly of trips made by Malian youths between the ages of 16 and 29 years travelling predominantly for work-related reasons (Fig. 2). This trip cluster is denoted as ‘youth workers’ (Fig. 2) since the cluster is dominated by work-related trips made by youths, although the clustering algorithm again included a smaller number of trips made by other groups. Travellers in the ‘youth worker’ cluster tended to make fewer trips than those in other trip clusters (p < 0.001)—a mean of 1.7 trips in the last year compared to 2.2 trips for travellers in the ‘women with children’ cluster and 2.8 trips for ‘general’ travellers not belonging to these two clusters. Trips in the ‘youth worker’ cluster were also significantly longer in duration (mean of 70 days *cf* 21 days for trips in other clusters, p < 0.001) and involved larger distances (mean of 185 km *cf* 130 km for trips in other clusters, p < 0.001). The vast majority of travellers in this group (87 %) had no children under the age of five.

The remaining trips belonged to a ‘general’ cluster. Travellers making trips in this ‘general’ cluster were predominantly male, belonged to all age groups, and trips in this cluster were for both family and work-related reasons (Fig. 2). In the Mali sample, many of the trips made by 16–29 year olds were absorbed by the ‘youth worker’ cluster and so travellers making trips in the ‘general’ cluster tended to be older than in the Burkina Faso, Zambia and Tanzania samples (mean of 44 years in the Mali sample *cf* 35 years in the samples from other countries, p < 0.001) and to have more children under 5 years (mean of 1.11 children in the Mali sample *cf* 0.56 children in the samples from other countries, p < 0.001). Travellers making trips in the ‘general’ cluster tended to make more overnight trips per year than those making trips in the ‘women with children’ and ‘youth worker’ clusters (mean of 3.5 trips *cf* 2.1 trips for other clusters, p < 0.001). Trips in the ‘general’ cluster also tended to cover larger distances than those in the ‘women with children’ cluster (mean of 192 km *cf* 136 km for the ‘women with children’ cluster, p < 0.001).

### Malaria risk factors

Trips in the ‘women with children’ cluster were significantly more likely to be to areas of high malaria transmission than those in the ‘general’ cluster in the samples from Mali (OR = 4.46, 95 % CI = 3.42–5.83), Burkina Faso (OR = 1.58, 95 % CI = 1.23–1.58), Zambia (OR = 1.50, 95 % CI = 1.20–1.89), and Tanzania (OR = 2.28, 95 % CI = 1.71–3.05) (Table 2). Travellers making trips in the ‘women with children’ cluster were also significantly more likely to own a bed net in the samples from Burkina Faso (OR = 1.77, 95 % CI = 1.25–2.53) and Zambia (OR = 1.74, 95 % CI = 1.34–2.27). In the Zambia sample, travellers making trips in the ‘women with children’ sample had a significantly higher perceived risk of malaria at their destination than those making trips in the ‘general’ cluster (OR = 1.35, 95 % CI = 1.03–1.77). In the Mali sample, trips in the ‘youth worker’ sample were significantly more likely to be to areas of high malaria transmission than those in the ‘general’ cluster (OR = 23, 95 % CI = 17–31).

**Table 2:** Results of logistic regression analyses for belonging to the ‘women with children’ and ‘youth worker’ traveller groups

		Mali (women with children)				Mali (youth workers)				Burkina Faso				Zambia				Tanzania			
		N	% WC	OR (CI)	P	N	% YW	OR (CI)	P	N	% WC	OR (CI)	P	N	% WC	OR (CI)	P	N	% WC	OR (CI)	P
Own bed net	No	–	–	–	–	–	–	–	–	236	20.8	–	–	435	22.3	–	–	122	21.3	–	–
	Yes	–	–	–	–	–	–	–	–	1026	31.7	1.77 (1.25–2.53)	0.001	1106	33.7	1.74 (1.34–2.27)	<0.001	1044	23.8	1.10 (0.69–1.79)	0.70
Used bed net on trip	No	–	–	–	–	–	–	–	–	903	27.9	–	–	1102	28.8	–	–	382	20.2	–	–
	Yes	–	–	–	–	–	–	–	–	359	34.0	1.29 (0.98–1.69)	0.064	439	34.9	1.20 (0.94–1.53)	0.14	784	25.3	1.36 (0.99–1.88)	0.064
Perceived malaria risk at destination	High	–	–	–	–	–	–	–	–	604	27.5	–	–	703	34.7	–	–	379	23.7	–	–
	Med	–	–	–	–	–	–	–	–	357	30.3	1.12 (0.87–1.61)	0.47	395	28.4	0.83 (0.63–1.09)	0.17	168	23.4	0.97 (0.71–1.34)	0.87
	Low	–	–	–	–	–	–	–	–	301	33.2	1.18 (0.87–1.61)	0.29	443	25.7	0.74 (0.56–0.97)	0.033	619	23.8	1.19 (0.76–1.84)	0.44
Malaria prevalence at destination	High	398	57.0	–	–	668	60.9	–	–	639	34.3	–	–	771	36.2	–	–	619	30.4	–	–
	Low	751	22.0	0.22 (0.17–0.29)	<0.001	660	10.2	0.04 (0.03–0.06)	<0.001	623	24.9	0.63 (0.49–0.81)	<0.001	770	24.8	0.66 (0.53–0.83)	0.004	547	15.9	0.44 (0.33–0.59)	<0.001
Own mobile phone	No	522	43.9	–	–	539	45.6	–	–	293	45.4	–	–	470	39.8	–	–	249	27.7	–	–
	Yes	627	26.0	0.50 (0.39–0.65)	<0.001	789	41.2	0.83 (0.62–1.11)	0.20	969	24.9	0.39 (0.30–0.52)	<0.001	1071	26.4	0.60 (0.47–0.76)	<0.001	917	22.5	0.81 (0.58–1.21)	0.19
Frequency of using mobile phone	Once per day or less	323	43.9	–	–	363	37.5	–	–	398	33.7	–	–	596	33.4	–	–	266	25.9	–	–
	Several times per day	304	22.0	0.67 (0.46–0.96)	0.030	426	44.4	1.37 (1.03–1.83)	0.033	571	18.7	0.49 (0.36–0.67)	<0.001	475	17.7	0.45 (0.33–0.60)	<0.001	651	21.0	0.75 (0.54–1.06)	0.088
Travelled with mobile phone	No	11	18.2	–	–	26	65.4	–	–	42	52.4	–	–	71	33.8	–	–	10	20.0	–	–
	Yes	616	26.1	1.85 (0.46–12.32)	0.44	763	40.4	0.34 (0.14–0.76)	0.011	927	23.6	0.35 (0.18–0.68)	0.003	1000	25.9	0.76 (0.46–1.30)	0.32	907	22.4	1.32 (0.32–8.99)	0.73
Share mobile phone	No	46	34.8	–	–	53	43.4	–	–	68	35.3	–	–	115	27.8	–	–	13	38.5	–	–
	Yes	581	25.3	0.64 (0.34–1.24)	0.17	736	41.0	0.90 (0.51–1.60)	0.71	901	24.1	0.78 (0.46–1.37)	0.35	956	26.3	0.93 (0.61–1.47)	0.79	904	22.2	0.42 (0.14–1.40)	0.13

The numbers of individuals, percentages belonging to each cluster (*WC* women with children, *YW* youth workers), and odds ratios (with 95 % confidence intervals) are shown

### Mobile phone ownership and usage

Travellers making trips in the ‘women with children’ cluster were significantly less likely to own a mobile phone than those making trips in the ‘general’ cluster in the samples from Mali (OR = 0.50, 95 % CI = 0.39–0.65), Burkina Faso (OR = 0.39, 95 % CI = 0.30–0.52) and Zambia (OR = 0.60, 95 % CI = 0.47–0.76) (Table 2). Of those who owned a mobile phone, travellers making trips in the ‘women with children’ cluster were significantly less likely to use their phone more than once per day compared to those making trips in the ‘general’ cluster in the samples from Mali (OR = 0.67, 95 % CI = 0.46–0.96), Burkina Faso (OR = 0.49, 95 % CI = 0.36–0.67) and Zambia (OR = 0.60, 95 % CI = 0.47–0.76). Furthermore, in the Burkina Faso sample, trips in the ‘women with children’ cluster were significantly less likely to involve people travelling with phones compared to trips in the ‘general’ cluster (OR = 0.35, 95 % CI = 0.18–0.68). Mobile phone sharing was relatively infrequent in all cases (less than 10 % of interviewees in all four country samples reported sharing their phone with others). In the Mali sample, travellers making trips in the ‘youth worker’ cluster showed no significant difference in mobile phone ownership compared to travellers making trips in the ‘general’ cluster; but were significantly more likely to use their phone more than once per day (OR = 1.37, 95 % CI = 1.03–1.83) and were significantly less likely to travel with their phone (OR = 0.34, 95 % CI = 0.14–0.76).

### Cross-border movement

The survey also captured cross-border movements that were not included in the within-country analysis (Table 3). Cross-border movements were recorded in all countries except for Tanzania and represented 4.8 % of all trips in the Mali sample, 4.7 % of all trips in the Burkina Faso sample and 3.2 % of all trips in the Zambia sample. International trips were predominantly made by male travellers while national trips were made by a smaller proportion of male travellers in the Burkina Faso sample (67 % male *cf* 51 % male for national trips, p = 0.011) and the Zambia sample (60 % male *cf* 38 % male for national trips, p < 0.001). In the Burkina Faso sample, international trips were more frequently made by travellers belonging to the 30–45 years old age group (54 % were 30–45 years *cf* 40 % for national trips, p = 0.010). International trips were more likely to be for work-related reasons than national trips in all country samples (52 % work-related *cf* 29 % for national trips, p < 0.001) and, in the Burkina Faso sample, although international trips were less likely to be made by travellers owning a bed net (67 % ownership *cf* 81 % for national trips, p = 0.007), international trips were more likely to be made by travellers using a bed net on their trip (41 % used bed net *cf* 29 % of national trips, p = 0.022). International trips were more likely to be made by travellers owing a mobile phone than national trips in all country samples (82 % ownership *cf* 63 % ownership for national trips, p < 0.001). There were no significant differences in terms of children accompanying travellers for international or national trips. International trips tended to involve longer durations than national trips in the Mali sample (mean of 64 days *cf* 37 days for national trips, p = 0.001) and the Burkina Faso sample (mean of 63 days *cf* 12 days for national trips, p < 0.001), however, they were relatively shorter in the Zambia sample (mean of 11 days *cf* 20 days for national trips, p < 0.001), possibly because several of the Zambian survey sites were border towns.

**Table 3:** Descriptive statistics for international travel from Mali, Burkina Faso and Zambia

	Mali		Burkina Faso		Zambia	
	*N*	% (CI)	*N*	% (CI)	*N*	% (CI)
Total	110		76		84	
Gender						
Female	41	37 (28–47)	25	33 (23–45)	34	40 (30–52)
Male	69	63 (53–72)	51	67 (55–77)	50	60 (48–70)
Age						
16–29	47	43 (33–53)	31	41 (30–53)	30	36 (26–47)
30–45	38	35 (26–44)	41	54 (42–65)	34	41 (30–52)
>45	25	23 (15–32)	4	5 (1–13)	19	23 (14–33)
Mean trip duration (range)		64.0 (1–350)		62.8 (1–365)		11.0 (1–90)
Purpose						
Work	47	43 (33–53)	49	64 (53–75)	43	51 (40–62)
Family	46	42 (32–52)	24	32 (21–43)	29	35 (24–46)
Other	15	14 (8–21)	2	2.6 (0.3–9.2)	11	13 (7–22)
Own bed net						
Yes	N/A	N/A	51	67 (55–77)	61	73 (62–82)
No	N/A	N/A	25	33 (23–45)	23	27 (18–38)
Use bed net on trip						
Always	N/A	N/A	30	39 (28–51)	19	23 (14–33)
Sometimes	N/A	N/A	0	0 (0.0–4.7)	3	3.6 (0.7–10.1)
Never	N/A	N/A	44	58 (46–69)	61	73 (62–82)
Own mobile phone						
Yes	80	73 (63–81)	69	91 (82–96)	70	83 (74–91)
No	30	27 (19–37)	6	8 (3–16)	12	14 (8–24)

The number of individuals (*N*) and percentage of the sample (exact binomial 95 % confidence interval), or mean (range) are shown. All interviewees did not answer all questions, hence there are some missing values

From the Mali survey sites, international travel was most commonly reported to other West African countries, such as Côte d’Ivoire, Guinea, Senegal, Burkina Faso, and Ghana, with occasional trips to France, the former colonial power, and Saudi Arabia for religious worship (Additional file 4: Figure S1), in agreement with the qualitative literature [31, 32]. From the Burkina Faso survey sites, international travel was most commonly reported to other West African countries, such as Côte d’Ivoire, Ghana, Benin, Togo, Mali, Senegal, and Niger. From the Zambia survey sites, international travel was most common to other East and Southern African countries, such as Malawi, Tanzania, South Africa, Namibia, Botswana, and the Democratic Republic of Congo. However, it should be noted that these destinations likely depend on the geographical distribution of the survey sites. Nevertheless, they reflect a high degree of informal cross-border movement, much of which is unlikely to be captured in international statistics on either migration or in air travel data.

## Discussion

Data on human movement patterns were collected and analysed in four countries in West, East and Southern Africa. The West African countries—Mali and Burkina Faso—are of interest because they are affected by seasonal agricultural labour movements [5, 15], while the Southern/East African countries—Zambia and Tanzania—have more comprehensive national transport networks [24] and youth movements due to boarding school [20]. Interestingly, despite differences in culture and geography, a cluster of trips was observed representing a group of travellers—women with children—that displayed very similar travel patterns across all four countries. In addition, a cluster of trips was observed representing a second major traveller group—youth workers—in the Mali data set, which corresponds very well with the qualitative literature [5, 15–17]. This is the first time that the movement patterns of these traveller groups have been quantified.

The ‘women with children’ trip cluster consists predominantly of trips made by women between the ages of 16 and 45 years who travel with children, usually for family-related reasons. These travellers tend to make fewer trips than other travellers and tend to travel shorter distances. In the samples from Mali, Burkina Faso, Zambia, and Tanzania, they were significantly more likely to travel to areas of high malaria prevalence, although in the Zambia sample, they had a higher perceived risk of malaria. In the Burkina Faso and Zambia samples, they were more likely to carry out malaria prevention measures, such as owning a bed net. Their travel to high prevalence areas is relevant to malaria transmission because the children that accompany them are more susceptible to malaria infection and may carry parasites with them back to the origin of travel. Considering the children that accompany them, this traveller group is expected to contribute greatly towards spatial malaria transmission. Additionally, some women who travel with children may also be pregnant, causing them to be more susceptible to malaria infection themselves, thus enhancing their contribution to spatial malaria transmission.

The Malian ‘youth worker’ trip cluster consists predominantly of trips made by young men and a significant number of women between the ages of 16 and 29 years who travel without children, usually for work-related reasons, on trips that last on the order of months. The lower age limit for this group corresponds to our eligibility criteria, and hence it is possible that travellers slightly younger than 16 years may also display similar travel patterns. These travellers tend to make fewer trips per year but their trips tend to involve longer distances compared to other traveller groups. They are also significantly more likely to travel to areas of high malaria prevalence, increasing their risk of contracting malaria and carrying it back to their origin of travel. The longer duration of their trips also provides more time for malaria transmission. It is of interest that this traveller group emerged from the analysis because it corresponds to a traveller group previously described only by qualitative research [5, 15–17]. It is expected that youth workers make a significant contribution to spatial malaria transmission in Mali because their movements correlate with seasonal rains and hence peak mosquito densities. The seasonality to these movements was not captured by the cluster analysis, possibly due to trip departure dates not corresponding with the seasonal definitions, however qualitative studies highlight the seasonal dimension of youth worker movements, as agricultural labour is highest during the rainy season when malaria is also most prevalent [5, 33], further highlighting the importance of this group to spatial malaria transmission in the Sahel.

With anonymous mobile phone signal data increasingly being used as a proxy for human movement patterns [2, 11], results from this survey on mobile phone usage behaviour can help quantify the biases inherent in this data source. In the samples from Mali, Burkina Faso and Zambia, travellers making trips in the ‘women with children’ cluster were significantly less likely to own a mobile phone than other travellers, and if they did own one, were significantly less likely to use it more than once per day. In the Burkina Faso sample, travellers making trips in the ‘women with children’ cluster were significantly less likely to travel with their phones compared to other travellers, but this trend was not seen in the other country samples. Phone sharing was relatively infrequent in all country samples (less than 10 % of interviewees claimed to share their phone), and no significant differences were seen between travellers making trips in the ‘women with children’ cluster and other travellers in terms of phone sharing, in contrast to previous results that rural women tend to share their phones more frequently [13]. Travellers making trips in the ‘youth worker’ cluster in Mali showed no significant difference in mobile phone ownership compared to travellers making trips in the ‘general’ cluster, but were significantly more likely to use their phones more than once per day and, surprisingly, were significantly less likely to travel with their phones. These results show that mobile phone usage behaviour is country-specific and hence mobile phone signal data may require country-specific corrections.

Several weaknesses of this study should be acknowledged when interpreting the results. The survey sites (Fig. 1) were a judgement/convenience sample chosen to: (a) capture a wide range of traveller groups; and, (b) take advantage of existing relationships with the local researchers. Since the study sites were not chosen randomly, inferences based on these results can not be assumed to be representative of each country as a whole, and instead represent the collection of study sites surveyed. Different locational biases were present in each country—in Tanzania, most of the survey sites were relatively urban, and in Burkina Faso, the survey sites were in or within the vicinity of the capital city, Ouagadougou. In Zambia, several of the survey sites were border towns, possibly explaining the short duration cross-border trips, and in Mali, the survey sites were relatively rural, which could partly explain the lower level of mobile phone ownership recorded there. The results analysed here provide a snapshot of traveller groups in a sub-set of each country, and could form a basis for larger-scale studies randomized at a national level. Cluster sampling could be implemented here by applying the principle of probability sampling to each national administrative level sequentially [34].

Other shortcomings include biases in the recording of trips and social desirability bias. Regarding trip recording, interviewees were asked in the questionnaire about their three most recent short-term and long-term trips (up to six in total). This could introduce a bias towards trips in the months preceding the interviews in two ways—recall bias, since recent trips may be easier to remember; and ‘trip clipping’ since, for people who have taken many trips, only the recent ones will be recorded. Furthermore, certain questions are subject to social desirability bias, since respondents may feel inclined to give the ‘right’ answer (e.g., questions on bed net and phone ownership and usage) [35].

## Conclusions

As a growing number of diseases are targeted for elimination, including poliomyelitis, Chagas disease and neglected tropical diseases, such as schistosomiasis, lymphatic filariasis and onchocerciasis, the role of travellers in sustaining transmission will become increasingly important. The combined survey and cluster analysis approach outlined here provides a powerful framework for identifying key traveller groups that, in combination with knowledge of the local epidemiology of disease transmission, will help to inform disease elimination programmes. Information on key traveller groups will also help to inform control programmes for emerging pathogens, such as Ebola [36], Zika [37], and pathogens yet to emerge [38].

A study following this approach randomized at a national scale is needed to infer nationally representative traveller groups, however, the survey results analysed here provide a snapshot of traveller groups present in a collection of sites in Mali, Burkina Faso, Zambia, and Tanzania. These provide preliminary evidence for the generality of the ‘women with children’ traveller group across all four countries, despite significant differences in culture and geography. Results from the Mali survey also provide preliminary evidence for youth workers as a key traveller group, in agreement with previous qualitative studies [5, 15–17].

Further survey work is encouraged, and questions recommended for inclusion in future DHS and Malaria Indicator Surveys to better characterise traveller groups are included in Additional file 1. However, given the expected contributions of these two groups to spatial malaria transmission—women with children due to the children they travel with having high parasite prevalence, and youth workers due to their movements being correlated with seasonal rains and peak mosquito densities—it is recommended that interventions aimed at interrupting spatial malaria transmission consider these groups. A number of interventions are available or being considered to address malaria importation including enhanced surveillance, targeted mass drug administration and provision of bed nets to travellers. Further studies utilizing field work, data analysis and mathematical models will help to develop a deeper understanding of the likely impact of these measures and to design optimal delivery strategies in a wide range of settings.

## Availability of supporting data

The data sets supporting the results of this article are available in Additional file 2.

## Additional files

10.1186/s12936-016-1252-3 Survey protocol, data and analysis. Details of the work-flow followed in each survey village/community, the sampling technique and data processing. Impact of survey timing on results. Questions recommended for inclusion in future Demographic and Health Surveys and Malaria Indicator Surveys to better characterise traveller groups.
10.1186/s12936-016-1252-3 Demographic and trip data for each survey country. Excel file containing two data sheets for each survey country—a traveller data sheet with a separate row for each interviewee and demographic variables as columns, and a trip data sheet with a separate row for each recorded trip and trip variables as columns. Trips are indexed with a unique TripID are matched to interviewees via a unique PersonID. Demographic and trip variables are summarized in “Traveller Variables” and “Trip Variables” sheets of the Excel file, respectively.
10.1186/s12936-016-1252-3 Descriptive statistics and trip clusters for the two Mali surveys.
10.1186/s12936-016-1252-3 International destinations recorded in surveys in Mali, Burkina Faso and Zambia.

## Abbreviations

DHS: Demographic and Health Survey
OR: odds ratio
