# Supplementary material for: Key traveller groups of relevance to spatial malaria transmission: a survey of movement patterns in four sub-Saharan African countries
Source: Malar J. 2016 Apr 12;15:200. doi: 10.1186/s12936-016-1252-3 (PMC4828820; doi:10.1186/s12936-016-1252-3)
Supplement: Supplementary file 1 — 10.1186/s12936-016-1252-3 Survey protocol, data and analysis. Details of the work-flow followed in each survey village/community, the sampling technique and data processing. Impact of survey timing on results. Questions recommended for inclusion in future Demographic and Health Surveys and Malaria Indicator Surveys to better characterise traveller groups. [file 12936_2016_1252_MOESM1_ESM.pdf]

Key traveller groups of relevance to spatial malaria transmission: A survey of movement patterns in four sub-Saharan African countries

John M. Marshall[1,2], Mahamoudou Touré[3], André Lin Ouédraogo[4,5], Micky Ndhlovu[6], Samson S. Kiware[7], Ashley Rezai[1], Emmy Nkhama[6], Jamie T. Griffin[1], T. Deirdre Hollingsworth[1,8], Seydou Doumbia[3], Nicodem J. Govella[7], Neil M. Ferguson[1], Azra C. Ghani[1]

[1]MRC Center for Outbreak Analysis and Modelling, Department of Infectious Disease Epidemiology, Imperial College London, London, UK
[2]Divisions of Biostatistics and Epidemiology, School of Public Health, University of California, Berkeley, California, USA
[3]Malaria Research and Training Center, University of Bamako, Bamako, Mali
[4]Centre National de Recherche et de Formation sur le Paludisme, Ouagadougou, Burkina Faso
[5]Institute for Disease Modeling, Bellevue, Washington, USA
[6]Chainama College of Health Sciences, Lusaka, Zambia
[7]Environmental Health and Ecological Sciences Thematic Group, Ifakara Health Institute, Dar es Salaam, Tanzania
[8]School of Life Sciences, University of Warwick, Warwick, UK

Additional file 1

1. Survey Protocol and Data

1.1. Survey Work Flow

We followed the following work flow in each of the survey villages/communities:

1. **Day 1:** Meeting with village/community leaders. Verify potential eligibility.
    a. Discuss the purpose of the survey, survey questions and sampling scheme with village/community leaders.
    b. When the survey team arrives, the village/community location is recorded and the equipment is tested.
    c. Initial information about population of the village (at least 240 adults) and times at which people are at home is obtained from village leaders.
    d. Verify number of households and check randomization scheme.
2. **Collection of data:**
    a. Days 2, 3 and 4:
        i. Each interviewer randomly selects households (see Sampling Technique below) to conduct interviews.
        ii. At each household, the interviewer identifies an individual who fulfils the eligibility criteria (16 years of age or older and reported making at least one overnight trip in the last year). If there is no individual (household member or visitor) at that location fulfilling the eligibility criteria, the next household

is sampled. If household members do fulfil the criteria but are not at home at the time of the survey, an attempt is made to find a time for a return visit.

iii. Obtain informed consent. The informed consent process for minors varied by country. In Mali and Tanzania, participants between 16-17 years of age were required to be accompanied by a relative of at least 18 years. In Burkina Faso, unmarried participants between 16-19 years were required to be accompanied by a relative of at least 20 years. In Zambia, participants between 16-18 years of age were required to be accompanied by a relative of at least 19 years. In all cases, the accompanying relative signed on their behalf after the participant has assented to participate.

iv. Conduct the interview.

b. Each interviewer interviews at least 10 individuals per day

c. At the end of each day, field teams back up the data without erasing the data that is held in the smarphone data collection app.

d. Continue until 240 individuals who have travelled have been surveyed in each survey village/community.

3. **Day 5:** Upload all village/community data at the host institution upon return.

1.2. Sampling Technique

We used a standard randomization technique whereby, from the center of each survey village, each interviewer chose a direction to work through the village by spinning a pen on a flat surface. In that direction, they chose every *n*th house, where *n* was a number between 2 and 5 chosen at random for each interviewer at the start of the survey. Each interviewer went in a direction such that the households surveyed did not overlap. For example, in the village sketch below, we show movement of a team which selects every 3rd house in orange. Houses are numbered from the center of the village out and back to the center again. In this example, households 3, 6, 9, 12, etc. (colored in red) would be included in the study.

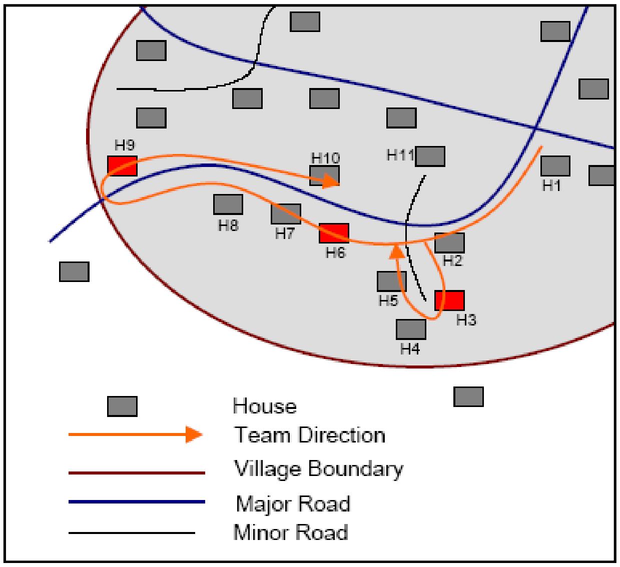

1.3. Data Processing

Survey results for each country were collected using the smartphone application EpiCollect [1] and uploaded to a server at Imperial College London. These results were then cleaned in the statistical computing program R and stored in demographic files, recording data pertaining to each individual interviewee, and trip files, recording data pertaining to each recorded trip (and linked to the corresponding demographic file via a person ID). The demographic and trip files are available along with a description of their columns in Additional file 2.

2. Analysis

2.1. Impact of Survey Timing on Results

To explore the impact of survey timing on results, we conducted the Mali survey during consecutive rainy and dry seasons. In total, 1,588 individuals were interviewed in Mali – 651 during the rainy season of September/October 2010 and 937 during the dry season of March 2011. Table S1 summarizes the demographics of those surveyed during each season. The rainy season sample had a significantly higher proportion of interviewees in the 16-29 year age group (51% in the rainy season sample c.f. 45% in the dry season, p=0.019). Interviewees in the rainy season sample also reported completing more overnight trips in the last year (mean of 1.94 trips in the rainy season sample c.d. 1.30 trips in the dry season sample, p<0.001). Differences in other descriptive statistics were statistically insignificant between the two samples.

A closer look at the distribution of trip clusters according to survey location and season suggests that the excess of interviewees in the 16-29 year age group in the rainy season sample and the higher number of trips reported in this sample could be due to the higher proportion of youth workers in this sample compared to the dry season sample. As Table S1 illustrates, the rainy season sample captured a significantly higher proportion of youth worker trips (41% of trips c.f. 28% of trips in the dry season sample, p<0.001). This excess of youth worker trips was concentrated in the farming villages of Boidie and Baraoueli (33% of trips in the “youth worker” cluster in the rainy season sample c.f. 14% of trips in the dry season sample, p<0.001) and the fishing villages of Carriere and Selenkegney (45% of trips in the “youth worker” cluster in the rainy season sample c.f. 12% of trips in the dry season sample, p<0.001). The difference between seasons in urban Bamako and Kalabancoro was not statistically significant. These observations are consistent with qualitative studies suggesting that youth workers are more present in rural areas during the rainy season when the bulk of the village’s agricultural work is done [2].

3. Questions recommended for inclusion in future Demographic and Health Surveys and Malaria Indicator Surveys

The results analysed here provide a snapshot of traveller groups present in a collection of sites in Mali, Burkina Faso, Zambia and Tanzania; however, a study following this approach at a national scale is needed to infer nationally representative traveller groups. Given that a stand-alone mobility survey is unlikely to be implemented at a national scale, we recommend the following questions for inclusion in future Demographic and Health Surveys (DHS) and Malaria Indicator Surveys (MIS) to better characterize key traveller groups. These are based on survey questions that were highly informative in the hierarchical cluster analysis for this study. Questions already included in some DHS/MIS surveys are listed in italics text:

Mobile phone questions:

1. *Do you own a mobile phone?*

If owns a mobile phone:

2. Are you the only person who uses your phone?
3. How often do you use your mobile phone to make or receive calls or text messages (once per day or less, several times per day)?

Trip-related questions:

4. *Where is your main place of residence (at the administrative level of commune or ward)?*
5. *In the last year, on how many separate occassions have you travelled away from your home community and slept away for at least one night?*

For the *x* most recent overnight trips:

6. Where did you spend most of your nights away (at the administrative level of commune or ward)?
7. How many nights did you go away for?
8. On which month did you leave to go on your trip?
9. What was the main purpose of your trip (work, family, other)?
10. Did any of your children accompany you on this trip?
11. If so, what were the ages of the children who went with you on this trip?
12. Did you use an insecticide-treated bed net while you were away on this trip (always, sometimes, never)?
13. Do you perceive there to be a risk of malaria where you traveled to (high, medium, low)?
14. If you own a mobile phone, did you take it on this trip?

4. References

1. Aanensen DM, Huntley DM, Feil EJ, Al-Own F, Spratt BG. EpiCollect: Linking smartphones to web applications for epidemiology, ecology and community data collection. PloS ONE. 2009; 4:e6968.
2. Remy G. Mobilite geographique et immobilisme sociale: Un exemple Volaique. Revue Tiers-Monde. 1977; 18:617-53.
