# Supplementary material for: Key traveller groups of relevance to spatial malaria transmission: a survey of movement patterns in four sub-Saharan African countries
Source: Malar J. 2016 Apr 12;15:200. doi: 10.1186/s12936-016-1252-3 (PMC4828820; doi:10.1186/s12936-016-1252-3)
Supplement: Supplementary file 3 — 10.1186/s12936-016-1252-3 Descriptive statistics and trip clusters for the two Mali surveys. [file 12936_2016_1252_MOESM3_ESM.pdf]

**Table S1 – Descriptive statistics and trip clusters for the two Mali surveys**

| **Interviewees:** | **September 2010:** | | **March 2011:** | |
|---|---|---|---|---|
| | ***N*** | **% (CI)** | ***N*** | **% (CI)** |
| **Total interviewees** | 651 | | 937 | |
| **Gender** | | | | |
| Female | 289 | 44 (41-48) | 441 | 47 (44-50) |
| Male | 359 | 55 (51-59) | 495 | 53 (50-56) |
| **Age** | | | | |
| 16-29 | 335 | 51 (48-55) | 425 | 45 (42-49) |
| 30-45 | 204 | 31 (28-35) | 307 | 33 (30-36) |
| >45 | 112 | 17 (14-20) | 205 | 22 (19-25) |
| **Mean # children <5 years (range)** | | 0.76 (0-6) | | 0.84 (0-9) |
| **Mean # trips (range)** | | 1.94 (1-51) | | 1.30 (1-51) |
| **Own phone** | | | | |
| Yes | 350 | 54 (49-58) | 509 | 54 (51-58) |
| No | 301 | 46 (42-50) | 426 | 45 (42-49) |
| **Trips:** | **September 2010:** | | **March 2011:** | |
| | ***N*** | **% (CI)** | ***N*** | **% (CI)** |
| **Total num. of trips** | 651 | | 937 | |
| **Cluster (all Mali survey locations)** | | | | |
| Women with children | 148 | 22 (19-25) | 250 | 23 (20-25) |
| Youth workers | 276 | 41 (37-45) | 311 | 28 (26-31) |
| General | 247 | 37 (33-41) | 537 | 49 (46-52) |
| **Cluster (Bamako and Kalabancoro)** | | | | |
| Women with children | 47 | 25 (19-32) | 43 | 17 (12-22) |
| Youth workers | 84 | 45 (38-52) | 133 | 51 (45-57) |
| General | 56 | 30 (23-37) | 84 | 32 (27-38) |
| **Cluster (Baraoueli and Boidie)** | | | | |
| Women with children | 50 | 22 (17-28) | 75 | 28 (23-34) |
| Youth workers | 76 | 33 (27-40) | 37 | 14 (10-18) |
| General | 101 | 44 (38-51) | 158 | 59 (52-64) |
| **Cluster (Baya)** | | | | |
| Women with children | 51 | 20 (15-25) | 79 | 27 (22-32) |
| Youth workers | 116 | 45 (39-51) | 35 | 12 (8-16) |
| General | 90 | 35 (29-41) | 183 | 62 (56-67) |
| **Cluster (Mopti and Fatoma)** | | | | |
| Women with children | N/A | N/A | 53 | 20 (15-25) |
| Youth workers | N/A | N/A | 106 | 39 (33-45) |
| General | N/A | N/A | 112 | 41 (35-47) |
