# Supplementary figures and images for: Key traveller groups of relevance to spatial malaria transmission: a survey of movement patterns in four sub-Saharan African countries
Source: Malar J. 2016 Apr 12;15:200. doi: 10.1186/s12936-016-1252-3 (PMC4828820; doi:10.1186/s12936-016-1252-3)

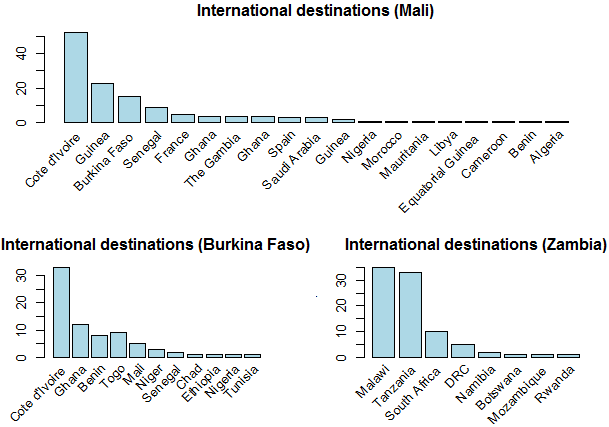

Supplement: Supplementary file 4 — 10.1186/s12936-016-1252-3 International destinations recorded in surveys in Mali, Burkina Faso and Zambia. [file 12936_2016_1252_MOESM4_ESM.tif]
